# Identification of circRNA Biomarker for Gastric Cancer through Integrated Analysis

**DOI:** 10.3389/fmolb.2022.857320

**Published:** 2022-03-10

**Authors:** Md. Tofazzal Hossain, Song Li, Md. Selim Reza, Shengzhong Feng, Xiaojing Zhang, Zhe Jin, Yanjie Wei, Yin Peng

**Affiliations:** ^1^ University of Chinese Academy of Sciences, Beijing, China; ^2^ Center for High Performance Computing, Joint Engineering Research Center for Health Big Data Intelligent Analysis Technology, Shenzhen Institutes of Advanced Technology, Chinese Academy of Sciences, Shenzhen, China; ^3^ Department of Statistics, Bangabandhu Sheikh Mujibur Rahman Science and Technology University, Gopalganj, Bangladesh; ^4^ Shenzhen Science & Technology Development Exchange Center, Shenzhen Science and Technology Building, Shenzhen, China; ^5^ Guangdong Provincial Key Laboratory for Genome Stability & Disease Prevention and Regional Immunity and Diseases, Department of Pathology, Shenzhen University School of Medicine, Shenzhen, China

**Keywords:** gastric cancer, circular RNA, computational approach, circRNA biomarker, circRNA-miRNA-gene interaction

## Abstract

Gastric cancer (GC) is one of the most common malignant tumors and ranks third in cancer mortality globally. Although, a lot of advancements have been made in diagnosis and treatment of gastric cancer, there is still lack of ideal biomarker for the diagnosis and treatment of gastric cancer. Due to the poor prognosis, the survival rate is not improved much. Circular RNAs (circRNAs) are single-stranded RNAs with a covalently closed loop structure that don’t have the 5′-3′ polarity and a 3′ polyA tail. Because of their circular structure, circRNAs are more stable than linear RNAs. Previous studies have found that circRNAs are involved in several biological processes like cell cycle, proliferation, apoptosis, autophagy, migration and invasion in different cancers, and participate in some molecular mechanisms including sponging microRNAs (miRNAs), protein translation and binding to RNA-binding proteins. Several studies have reported that circRNAs play crucial role in the occurrence and development of different types of cancers. Although, some studies have reported several circRNAs in gastric cancer, more studies are needed in searching new biomarkers for gastric cancer diagnosis and treatment. Here, we investigated potential circRNA biomarkers for GC using next-generation sequencing (NGS) data collected from 5 paired GC samples. A total of 45,783 circRNAs were identified in all samples and among them 478 were differentially expressed (DE). The gene ontology (GO) analysis of the host genes of the DE circRNAs showed that some genes were enriched in several important biological processes, molecular functions and cellular components. The Kyoto encyclopedia of genes and genomes (KEGG) pathway analysis revealed that some host genes were enriched in several GC related pathways. The circRNA-miRNA-gene interaction network analysis showed that two circRNAs circCEACAM5 and circCOL1A1 were interacted with gastric cancer related miRNAs, and their host genes were also the important therapeutic and prognostic biomarkers for GC. The experimental results also validated that these two circRNAs were DE in GC compared to adjacent normal tissues. Overall, our findings suggest that these two circRNAs circCEACAM5 and circCOL1A1 might be the potential biomarkers for the diagnosis and treatment of GC.

## Introduction

Gastric cancer (GC) is the fifth most common malignant tumors and third leading cause of cancer related deaths worldwide (Bray et al., 2018). Despite of having advanced diagnosis and treatment strategies, the prognosis of gastric cancer is still poor, and a 5-year survival rate is still less than 30% (Allemani et al., 2015). Due to poor prognosis, most of the patients are diagnosed at advance stages and are not capable of receiving the surgical therapy (Karimi et al., 2014). Therefore, new molecular diagnostic biomarker and therapeutic targets are of great interest to understand the molecular mechanism of gastric cancer diagnosis and treatment for early detection of patients and improving the overall survival rate.

Circular RNAs (circRNAs) are a class of single-stranded RNAs formed by a covalently closed loop structure without 5′-3′ polarities or 3′ polyA tail (Jiao Li et al., 2020). Although most of the circRNAs are generated from back splicing of exons, some are generated from introns and some are from both of exons and introns (Wang and Dong, 2019). Because of the circular structure, circRNAs are more stable than linear RNAs, and are abundant in different species. CircRNAs have the vast potentiality to be a molecular biomarker for diagnosis, treatment and prognosis of different cancers. Previous studies have demonstrated that circRNAs are involved in several biological processes including cell cycle, proliferation, apoptosis, autophagy, migration and invasion, in different types of cancers (Han et al., 2017; Sollott, 2017; Meng et al., 2018; Yu et al., 2018; Zhang et al., 2018). In recent years, circRNAs have been found to participate in several molecular mechanism like sponging microRNAs (miRNAs), protein translation and binding to RNA-binding proteins (Hansen et al., 2013; Kristensen et al., 2018; Chen et al., 2019; Zang et al., 2020). Moreover, some circRNAs can bind protein/peptides (Legnini et al., 2017; Pamudurti et al., 2017). Several studies have identified that circRNAs play key role in the occurrence and progression of a lot of malignancies like glioma (Li et al., 2018), hepatocellular carcinoma (Han et al., 2017), pancreatic carcinoma (Huang et al., 2018), gastric cancer (Chen et al., 2017), colon cancer (Xu et al., 2017), prostate cancer (Tucker et al., 2020), breast cancer (Li et al., 2019) etc. Despite several circRNAs have been reported in GC, more studies are needed to identify new biomarker for the diagnosis and treatment of this cancer.

In this study, to identify potential circRNA biomarker, we used next-generation sequencing (NGS) data from gastric cancer tissue and adjacent normal tissues. Then, differentially expressed circRNAs were identified and circRNA-miRNA-gene interaction network was constructed. GO term and KEGG pathway analyses were performed for the host genes of the DE circRNAs. GO term analysis showed that several genes functioned in some biological processes, molecular functions and cellular components. The KEGG pathway analysis showed that some genes were involved in several gastric cancer related pathways. We found two circRNAs circCEACAM5 and circCOL1A1 which were differentially expressed in all samples and these circRNAs were highly interacted with gastric cancer related miRNAs. In addition, the host genes (CEACAM5 and COL1A1) of these circRNAs were the important therapeutic and prognostic biomarker for gastric cancer (Hu and Chen, 2012; Zhou et al., 2015; Zhaoxing Li et al., 2020). The important properties of circRNAs includes sponging miRNAs and regulating gene transcription. As the host genes of the two circRNAs were important biomarker of GC and interacted with GC related miRNAs, they might have the potential to be important biomarker for GC. The expression of these two circRNAs were also validated by qRT-PCR experiments. Our results suggest that the circRNAs circCEACAM5 and circCOL1A1 might be the potential biomarkers for the diagnosis and treatment of GC.

## Methods and Materials

### Sample Collection

Fifteen pairs (10 for RT-PCR and 5 for RNA sequencing) of non-neoplastic gastric tissues (NT) and GCs tissues from Shenzhen Second People’s Hospital (The First affiliated Hospital of Shenzhen University) were examined in the study. All tissues received no radiotherapy or chemotherapy before surgery and stored in RNAlater immediately after surgery. All patients provided written informed consent, approved by the ethics committee of Shenzhen University School of medicine.

### Ribonucleic Acid Library Preparation

Total RNA was extracted from 5 paired GC samples. Ribosomal RNA was digested using ribo zero kit and linear RNA was removed using rnase R. CircRNA was fragmented and cDNA was synthesized with six base random primers and purified. Strand-specific library is prepared via incorporating a chemical label deoxy-UTP (dUTP) during production of the second-strand cDNA. The second-strand cDNA was specifically digested by UNG enzyme. The constructed library was analyzed by Agilent 2,100 Bioanalyzer and was sequenced on an Illumina Hiseq 2,500 (Chi Biotech, Shenzhen).

### Ribonucleic Acid-Seq Data Analysis

We used 10 samples from 5 gastric cancer patients to investigate potential circRNA biomarker. From each patients, two samples were collected: one from the cancer tissue and other from the adjacent normal tissue. After getting the raw data in fastq format, the quality control analysis was performed. The quality control was done using the NGSQCToolkit (Patel and Jain, 2012) and the quality score 20 was used as the cutoff point. Filtering/trimming was carried out if the quality of the reads failed to reach the cutoff point. The fastq reads were aligned to the human reference genome (version GRCh38) using the BWA (Li and Durbin, 2009) aligner. CircRNAs were identified using the software CIRI (version 2) (Gao et al., 2015). The identified circRNAs were then annotated with the gene annotation file corresponding to the reference genome and the full length circRNA sequences were extracted. The full length circRNA sequences for all circRNAs were considered as the reference genome and the fastq reads were mapped using the bowtie2 (Langmead and Salzberg, 2012) aligner. Then count data was generated using bedtools (Quinlan and Hall, 2010) multiBamCov with the output of bowtie2 (converted to bam, sorted and indexed). R package DESeq (Anders and Huber, 2010) and DESeq2 (Love et al., 2014) were used to identify differentially expressed (DE) circRNAs for individual patient and combining all patients together respectively. |Log2FoldChange|>1 and *p*-value<0.05 were considered as the cut-off for defining significant DE circRNAs. For the DE analysis of combination of all patients, we also considered false discovery rate (FDR) at 10%.

### Real-Time-Polymerase Chain Reaction and Real-Time Quantitative Polymerase Chain Reaction

According to the manufacturer’s protocol, total RNA was extracted using Trizol reagent (251,808, Invitrogen). Reverse transcription and real-time PCR were performed using GoScript™ Reverse Transcription Mix (A2800 and A6002, Promega). Divergent primers were designed. All primers were synthesized by Sangon Biotech. Results were normalized to the 18S mRNA in each sample. We used the following primer sequences.

**Table:** 

circCEACAM5	F	CTCAGCTGGGGCCACTG
	R	GTGTCCGGCCCATCAGTC
**circMISP**	F	GGC​AGT​TAC​TCG​GTG​TCT​GA
	R	GGT​ATC​TGG​TCA​CGC​GGT​C
**circCOL1A1**	F	CTG​GCA​GCC​CTG​GTC​CTG​AT
	R	ATC​TGC​GCC​AGG​GAA​ACC​AC
**circLIPF**	F	AAC​ACG​AGT​CGC​TTG​GAT​GT
	R	TGC​CAT​TGT​TAA​AAG​CAG​CCA
**circPGC**	F	GAT​GAG​GCC​ACC​ACA​GCT​A
	R	TGG​ATG​CTC​TGG​ACC​TGC​T

### CircRNA-miRNA-Gene Interaction Network Analysis

We were interested with those circRNAs which were DE in four or more patients (out of five patients). Then, some top DE circRNAs were selected for miRNA interaction analysis. The circRNA-miRNA interaction was predicted using miRanda (Enright et al., 2003) software. The miRNA sequences were downloaded from mirbase (Kozomara et al., 2019). From the circRNA-miRNA interaction, a sub-network was constructed keeping only the gastric cancer related miRNAs. Then a circRNA-miRNA-gene network was constructed using Cytoscape (Shannon et al., 2003). The genes were the host genes of the circRNAs. Finally, a sub-network was constructed with the top 5 hub circRNAs.

### Gene Ontology Term and Kyoto Encyclopedia of Genes and Genomes Pathway Analysis

Search Tool for the Retrieval of Interacting Genes (STRING; http://string-db.org/cgi/input.pl) is a database for obtaining the protein-protein interaction of the provided genes. The host genes of the DE circRNAs were mapped to STRING database, and the protein-protein interaction among the genes was obtained. Then, a PPI network was constructed using the software Cytoscape, and from the network, the top 50 hub genes were selected for GO term and KEGG pathway enrichment analyses. With the selected hub genes, GO term and KEGG pathway enrichment analyses were performed using DAVID (Huang et al., 2009) to know the function of the circRNAs. The threshold *p*-value<0.05 was used for the significance of the enrichment analysis.

## Results

### Expression Profiles of circRNAs

**FIGURE 1:**
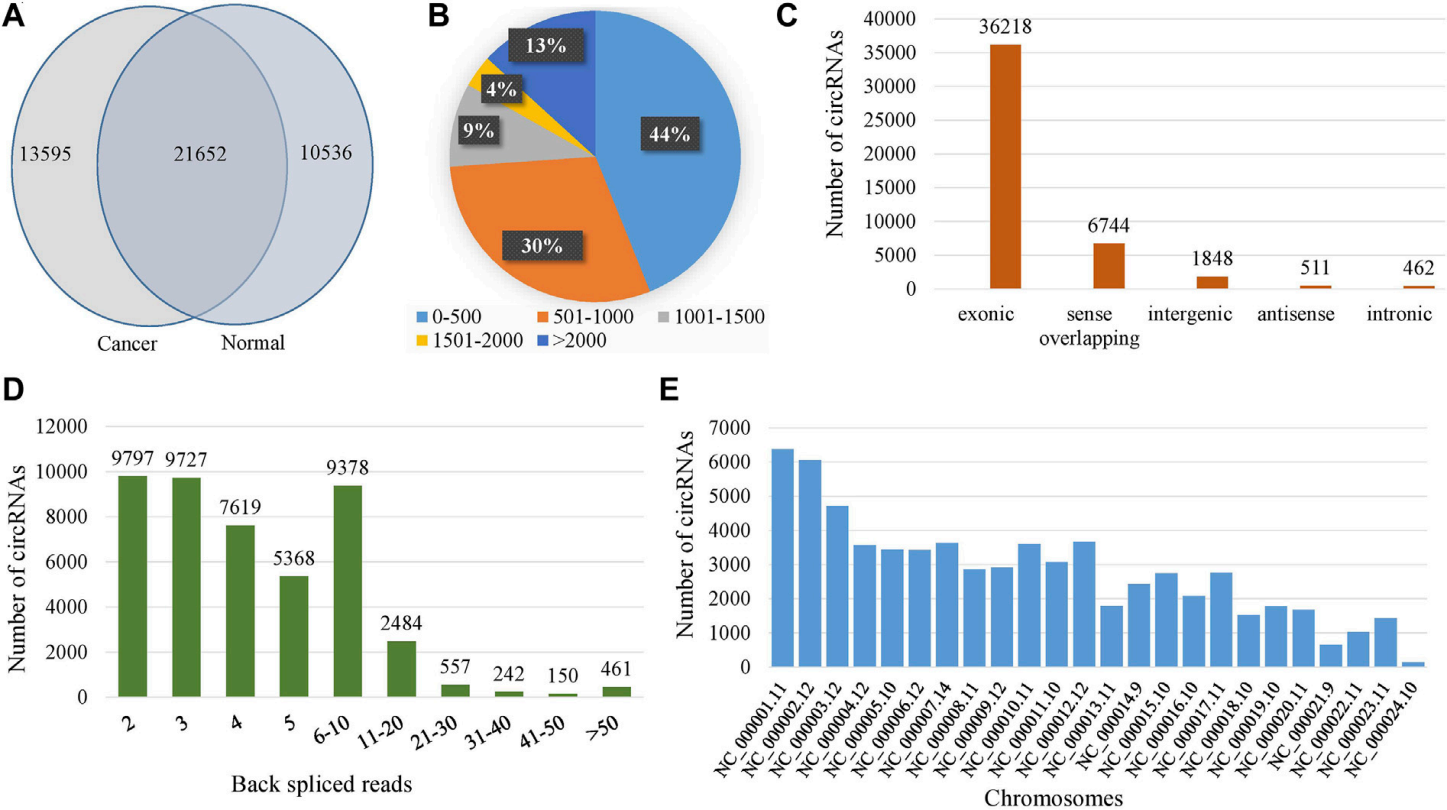
Expression profiles of circRNAs. **(A)** Venn-diagram of the identified circRNAs in normal and cancer samples, **(B)** Length distribution of circRNAs, **(C)** Classification of the identified circRNAs, **(D)** Distribution of the number of back spliced reads, and **(E)** Chromosome distribution of the identified circRNAs.

A total of 45,783 circRNAs were identified in all samples and 21,652 of them were co-expressed in cancer and normal samples (Figure 1A). Among the total circRNAs, 79% (36,218/45,783) were exonic, 1% (462/45,783) were intronic, 4% (1848/45,783) were intergenic, 15% (6,744/45,783) were sense overlapping and 1% (511/45,783) were antisense (Figure 1C). The length distribution of the circRNAs was shown in Figure 1B and observed that the length of 74% (44 + 30) of the circRNAs was less than 1,000 nucleotides (nt). The length of only 13% circRNAs was greater than 2000 nt. The number of back-spliced reads for the 97% of the circRNAs were less than 20 (Figure 1D). The average number of back spliced reads per circRNA was 6 whereas the minimum and the maximum number of back spliced reads were 2 and 898 respectively. The distribution of circRNAs in different chromosomes were heterogeneous, most circRNAs were originated from chromosome NC_000,001.11 (chromosome 1) (Figure 1E). The average number of circRNAs per chromosome was 1,405. The length of the circRNAs ranged from 48 to 199,579 nt while median and average lengths were 563 nt and 3,042 nt respectively (Table 1).

**TABLE 1:** Summary Statistics of the circRNAs.

Summary statistics	
Min	48
1st Quartile	359
Median	563
Average	3,042
3rd Quartile	1,037
Max	199,579

**FIGURE 2:**
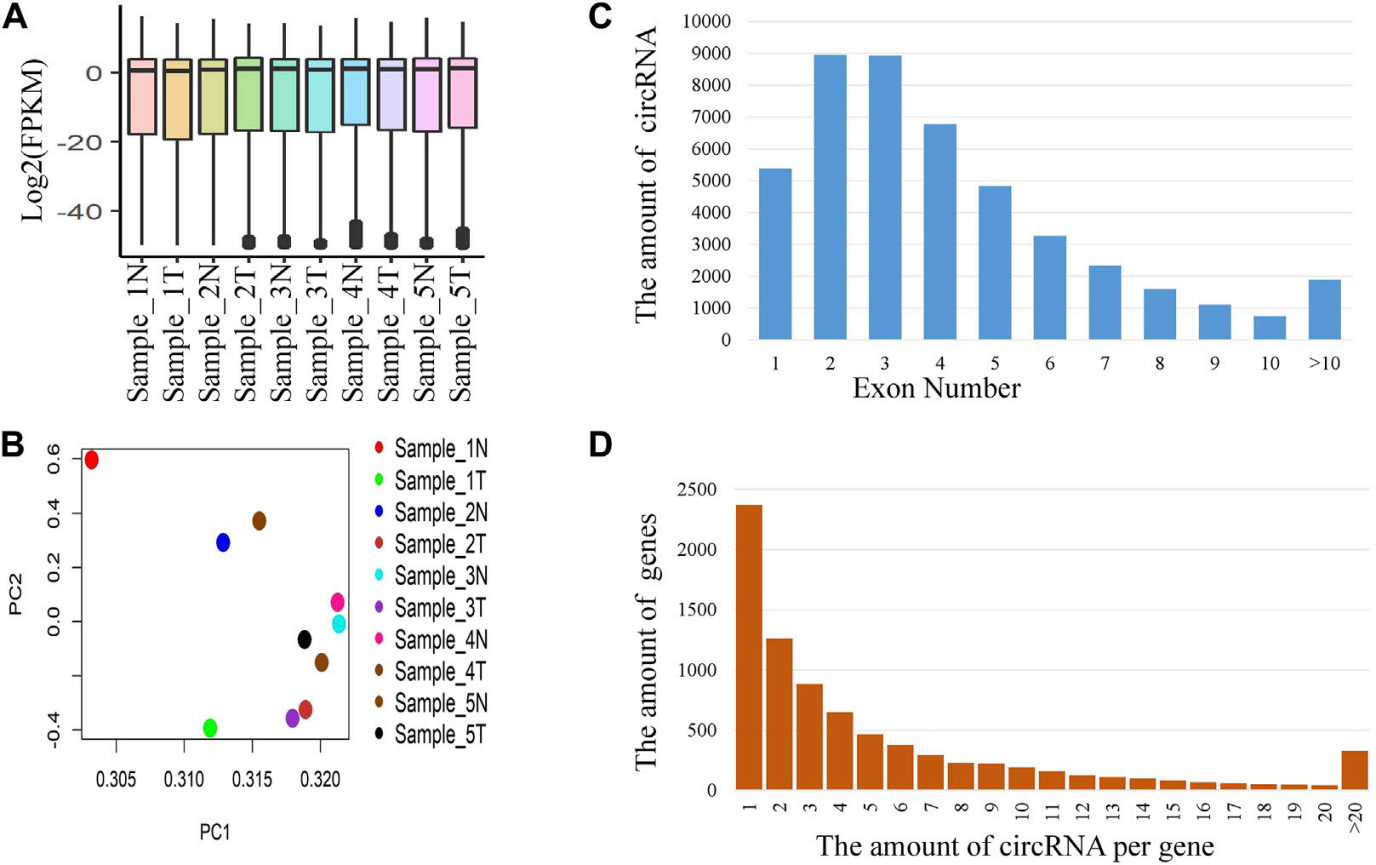
Distribution of circRNAs. **(A)** Expression (FPKM) of circRNAs for different samples, **(B)** Biplot of PCA loadings for different samples, **(C)** Distribution of circRNAs derived from different number of exons, and **(D)** Distribution of the number of circRNAs per gene.

The expression values of the 10 samples from the five patients were shown in Figure 2A. The expression values were almost homogeneous across the samples. We also performed the Principal Component Analysis (PCA) of the expression values and the loading of the PC1 and PC2 were shown in Figure 2B. The distribution of circRNAs across the number of exons was shown in Figure 2C. Majority of the circRNAs were composed of 2 or 3 exons. About 76% of the circRNAs were derived from less than or equal to 5 exons. The distribution of the number of circRNAs per gene was given in Figure 2D. Total 45,783 circRNAs were originated from 8,095 genes. Majority of the cases (about 30%), the number of circRNA per gene was 1. About 55% of the cases, the number of circRNAs per gene was less than or equal to 3.

### Differential Expression Analysis of circRNAs

**FIGURE 3:**
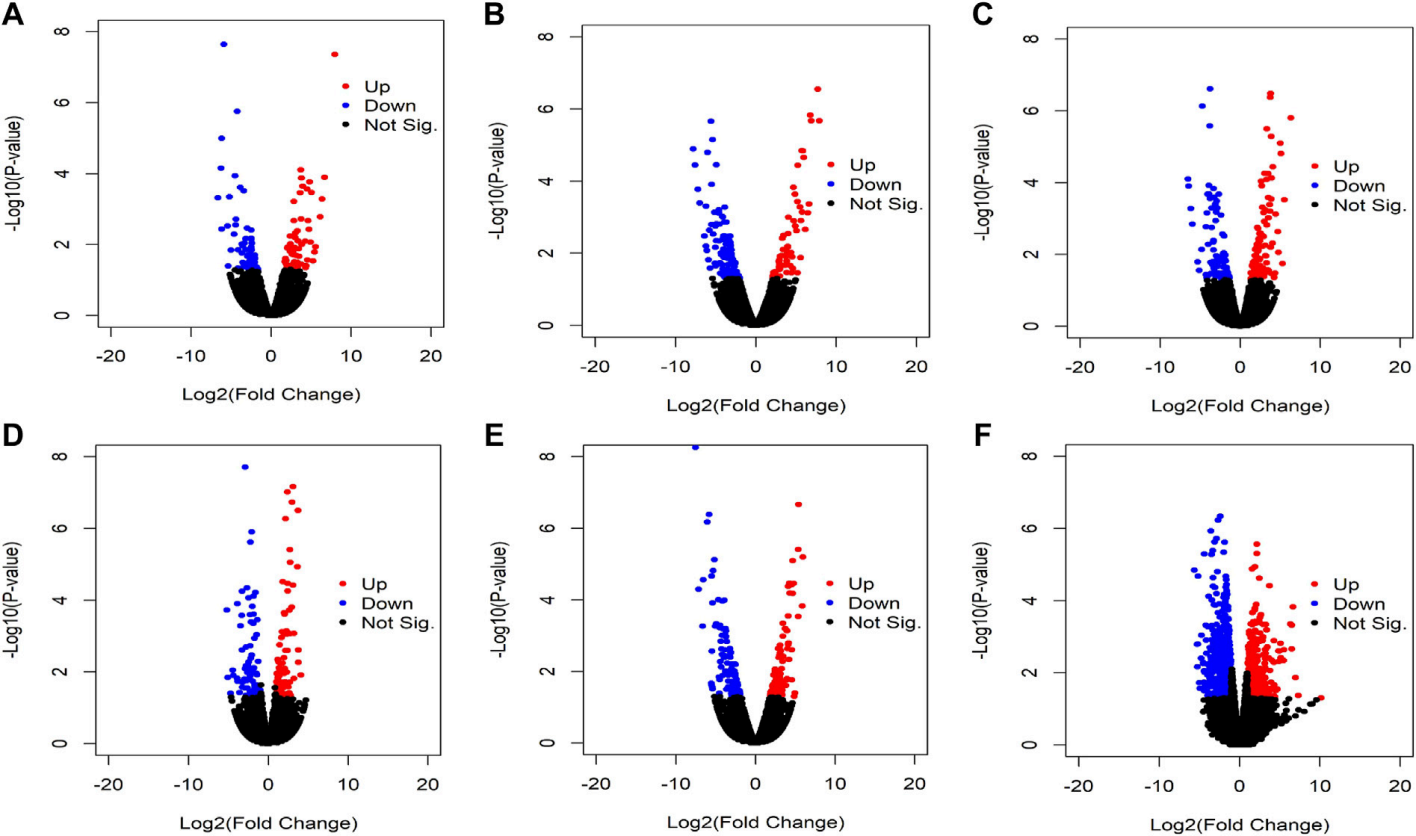
Volcano plot of the circRNAs. Red colors indicate upregulated circRNAs while blue colors indicate downregulated circRNAs and black colors indicate not significant. Results of DE analysis of **(A)** Sample_1N vs Sample_1T, **(B)** Sample_2N vs Sample_2T, **(C)** Sample_3N vs Sample_3T, **(D)** Sample_4N vs Sample_4T, **(E)** Sample_5N vs Sample_5T, and **(F)** Sample_N vs Sample_T.

**FIGURE 4:**
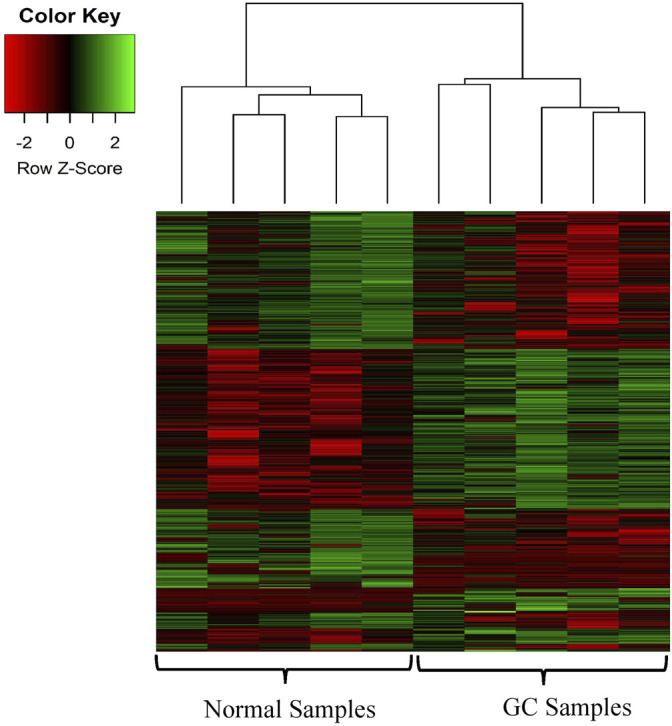
Heatmap of the DE circRNAs. The heatmap represents the expression profile of the DE circRNAs in GC compared to the adjacent normal tissues. The color scale indicates the Log2FoldChange of the expression value for each circRNA in cancers vs normal tissues. Red and green colors indicate down-regulation and up-regulation of the circRNAs respectively.

We identified the differentially expressed circRNAs between two groups (normal vs cancer). The R package DESeq was used to find the differentially expressed circRNAs. We collected 10 samples from five patients: for each patients two samples were collected one from cancer tissue and other from the adjacent normal tissue. We also performed DE analysis between normal and cancer groups for each patient. The volcano plot was used to find the target circRNAs with the thresholds *p*-value<0.05 and |log2FoldChange| >1 (Figure 3). The number of DE circRNAs for all samples were given in Table 2. Out of 45,783 total circRNAs, 478 were differentially expressed between normal vs cancer groups. From Table 2, we observed that about half of the DE circRNAs were upregulated, whereas the other half were downregulated. The expression patterns of these circRNAs were shown in the heatmap (Figure 4). The dendrograms showed that the cancer and normal samples were clearly distinguishable. Firstly, we identified DE circRNAs for all patients (5 normal samples vs 5 cancer samples) and performed DE analysis for each individual patient and isolated those circRNAs which were DE in four or more patients. 4 upregulated and 7 downregulated circRNAs were found to be DE in more than four patients. We selected 8 circRNAs (4 upregulated and 4 downregulated) from the DE analysis of individual patient. These 8 circRNAs were not only DE patient-wise but also DE while performed DE analysis combining all patients (5 normal samples vs 5 cancer samples). Finally, these 8 circRNAs were selected for circRNA-miRNA interaction analysis. The details of the 8 circRNAs were given in Table 3.

**TABLE 2:** Number of DE circRNAs in different samples.

Control	Case	Upregulated	Downregulated	Total
Sample_1N	Sample_1T	91	90	181
Sample_2N	Sample_2T	90	114	204
Sample_3N	Sample_3T	126	100	226
Sample_4N	Sample_4T	94	89	183
Sample_5N	Sample_5T	136	139	275
Sample_N	Sample_T	242	236	478

**TABLE 3:** Top 8 (4 upregulated and 4 downregulated) circRNAs.

circRNA name	circRNA ID	Base mean	Log2 FC	*p*-value	FDR	Host gene
circCEACAM5	NC_000,019.10:41,720,921|41,727,352	163.53	3.05	3.9 × 10^−03^	1.0 × 10^−01^	CEACAM5
circMISP	NC_000,019.10:756,889|760,039	2093.45	2.42	3.4 × 10^−03^	9.0 × 10^−02^	MISP
circCOL1A1	NC_000,017.11:50,194,129|50,194,828	540.5	2.66	2.4 × 10^−10^	1.7 × 10–^06^	COL1A1
circCOL3A1	NC_000,002.12:188,999,860|189,004,054	2,678.48	1.92	8.7 × 10^−07^	4.3 × 10^−04^	COL3A1
circLIPF	NC_000,010.11:88,667,286|88,675,657	1766.22	-4.50	8.8 × 10^−03^	1.0 × 10^−01^	LIPF
circPGC	NC_000,006.12:41,741,795|41,742,489	2,300.96	-3.48	3.7 × 10^−03^	1.0 × 10^−01^	PGC
circPGC_1	NC_000,006.12:41,742,289|41,743,389	18,599.68	-3.58	1.3 × 10^−03^	5.6 × 10^−02^	PGC
circPGC_2	NC_000,006.12:41,740,207|41,740,610	401.54	-3.67	4.3 × 10^−06^	1.3 × 10^−04^	PGC

### CircRNA-miRNA-Gene Interaction Analysis

**FIGURE 5:**
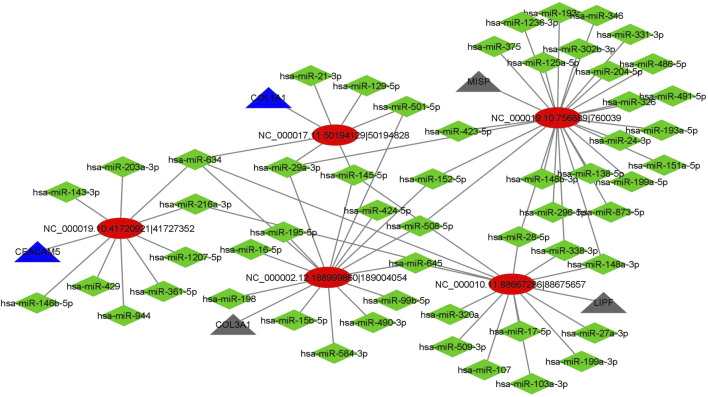
CircRNA-miRNA-gene interaction network. Ellipse shape indicates circRNA, diamond shape indicates miRNA while triangle indicates gene. Blue triangle indicates genes related to GC and black triangle indicates other genes.

We got a total of 478 circRNAs as DE between normal vs cancer groups. Among these circRNAs, we investigated those which were DE in more number of patients. We chose those circRNAs for interaction analysis, which were DE in four or more patients (out of five patients). We selected the 4 upregulated and 4 downregulated (top 4 out of 7 downregulated) circRNAs for miRNA interaction analysis. We got a total of 1990 interactions with 8 circRNAs and 1,278 miRNAs. The miRNA sequences were downloaded from the mirbase database. From the online search we found 64 GC related miRNAs out of 1,278 miRNAs in the network. Next, we extracted the interactions of these 64 miRNAs and obtained a total of 81 interactions. Then, a circRNA-miRNA-gene network was constructed using Cytoscape. Here, the genes were the host genes of the circRNAs. Finally, we constructed a subnetwork with top 5 hub circRNAs (Figure 5). From the network, we observed that two genes were functionally related to GC. Two circRNAs circCEACAM5 and circCOL1A1 were interacted with GC related miRNAs and their host genes were also related to GC. This indicated that these two circRNAs might be potential biomarker for GC. The detail list of top 5 hub circRNAs, their interacted GC related miRNAs and their host genes were given in Table 4.

**TABLE 4:** The five hub circRNAs, their interacted gastric cancer related miRNAs and the host genes of the circRNAs.

Name	circRNA ID	Host gene	Interacted miRNAs
CircMISP	NC_000,019.10:756,9889|760,9039	MISP	hsa-miR-508-5p, 873-5p, 125a-5p, 1236-3p, 138-5p, 148b-3p, 148a-3p, 151a-5p, 152-5p, 193a-5p, 193a-3p, 199a-5p, 204-5p, 24-3p, 28-5p, 296-5p, 29a-3p, 302b-3p, 326, 331-3p, 338-3p, 346, 375, 423-5p, 486-5p, 491-5p
CircCOL3A1	NC_000,002.12:188,999,860|189,004,054	COL3A1	hsa-miR-634, 584-3p, 645, 99b-5p, 145-5p, 152-5p, 15b-5p, 16-5p, 198, 195-5p, 29a-3p, 424-5p, 490-3p, 501-5p, 508-5p
CircLIPF	NC_000,010.11:88,667,286|88,675,657	LIPF	hsa-miR-509-3p, 634, 645, 103a-3p, 107, 145-5p, 148a-3p, 17-5p, 199a-3p, 216a-3p, 28-5p, 27a-3p, 320a, 338-3p
CircCEACAM5	NC_000,019.10:41,720,921|41,727,352	CEACAM5	hsa-miR-634, 944, 1207-5p, 143-3p, 146b-5p, 203a-3p, 216a-3p, 361-5p, 429
CircCOL1A1	NC_000,017.11:50,194,129|50,194,828	COL1A1	hsa-miR-634, 129-5p, 145-5p, 21-3p, 29a-3p, 501-5p

### Differential Expression Analysis of the Targeted miRNAs Genes and Candidate circRNAs

**FIGURE 6:**
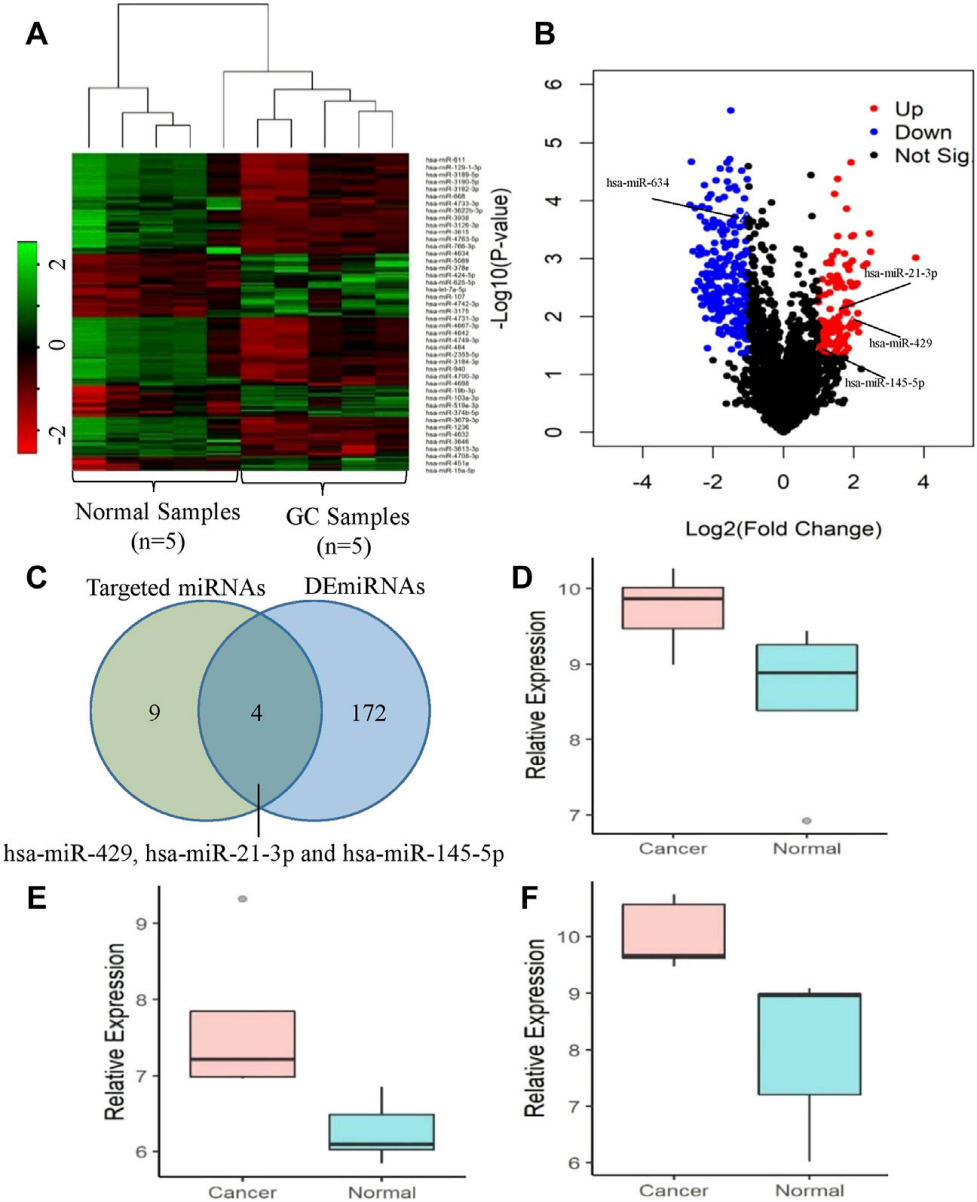
Expression analysis of the targeted miRNAs of the circRNAs circCEACAM5 and circCOL1A1. **(A)** Clustered heatmap represents the expression profile of the DE miRNAs in GC compared to normal tissues. The color scale indicates the Log2FoldChange of the expression value for each miRNA in cancers vs normal tissues. Red color implies down-regulation while green color indicates up-regulation. **(B)** Volcano plot of the DE miRNAs in GC relative to normal tissues. Red, blue and black dots indicate up-regulation, down-regulation and not significant respectively. **(C)** Identification of the potential target miRNAs of the circRNAs circCEACAM5 and circCOL1A1. Expression levels of **(D)** hsa-miR-429, **(E)** hsa-miR-21-3p and **(F)** hsa-miR-145-5p in GC compared to normal tissues.

We performed the differential expression analysis of the miRNAs in gastric cancer tissue and the adjacent normal tissue using the GEO dataset (GSE158315). R package limma (Ritchie et al., 2015) was used to find the DE miRNAs. The expression patterns of the DE miRNAs were shown in Figure 6A. The dendrograms showed that the cancer and normal samples were clearly distinguishable. The volcano plot (Figure 6B) was used to find the DE miRNAs with the threshold *p*-value<0.05 and |log2FoldChange|>1. We found a total of 176 DE miRNAs of which 60 were upregulated and 116 were downregulated. The number of targeted miRNAs for circRNAs circCEACAM5 and circCOL1A1 were 9 and 5 respectively. The unique targeted miRNAs for these two circRNAs were 13 (Figure 6C). MiRNAs hsa-miR-634 and hsa-miR-429 were common between the 9 targeted miRNAs of circCEACAM5 and the 176 DE miRNAs. Again, miRNAs hsa-miR-634, hsa-miR-21-3p and hsa-miR-145-5p were common between the 5 targeted miRNAs of circCOL1A1 and the 176 DE miRNAs. MiRNAs hsa-miR-429, hsa-miR-21-3p and hsa-miR-145-5p were found to be significantly upregulated in GC (Figures 6D–F). Therefore, miRNA hsa-miR-429 might be the potential target of the circRNA circCEACAM5, and miRNAs hsa-miR-21-3p and hsa-miR-145-5p might be the potential targets of the circRNA circCOL1A1.

**FIGURE 7:**
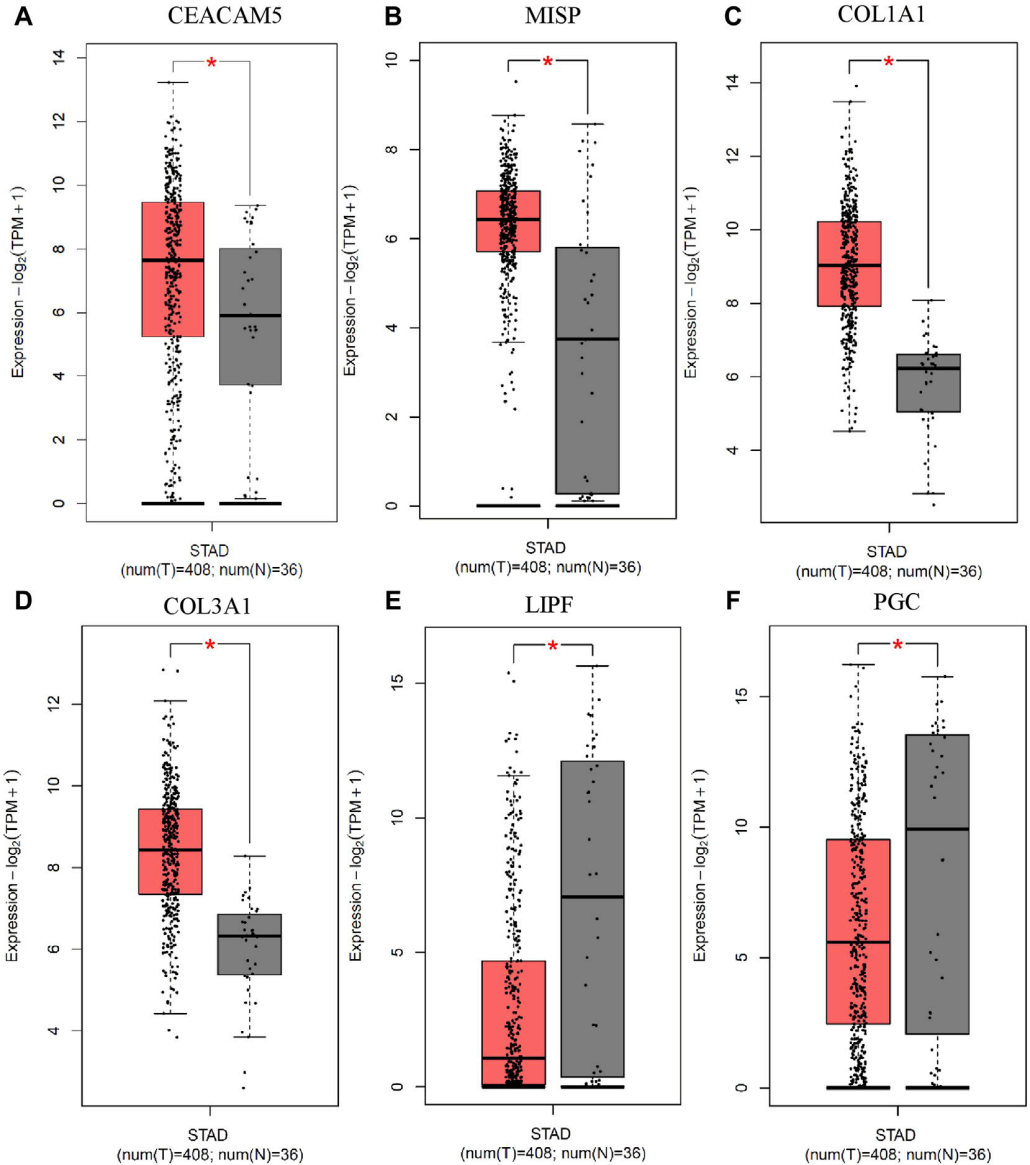
Validation of the expression of 6 host genes of the top up and down regulated circRNAs in GC. The expression of **(A)** CEACAM5, **(B)** MISP, **(C)** COL1A1 and **(D)** COL3A1 was upregulated, and the expression of **(E)** LIPF and **(F)** PGC was downregulated in gastric tumor compared to normal samples from the TCGA data. The cutoffs |Log2FoldChange|>1 and *p*-value<0.05 were considered as statistically significant.

We selected top 8 circRNAs (4 upregulated and 4 downregulated) for the downstream analysis. These 8 circRNAs were originated from 6 host genes. The expression of these 6 host genes were checked by the TCGA data of 408 gastric tumor samples and 36 normal samples obtained from Gepia (version 2) (Tang et al., 2019). We found that 4 genes were upregulated in the GC tissue compared to the normal tissue Figures 7A–D. In addition 2 genes were downregulated in GC compared to the normal samples Figures 7E,F. The 4 upregulated host genes might be the potential targets of their respective circRNAs.

**FIGURE 8:**
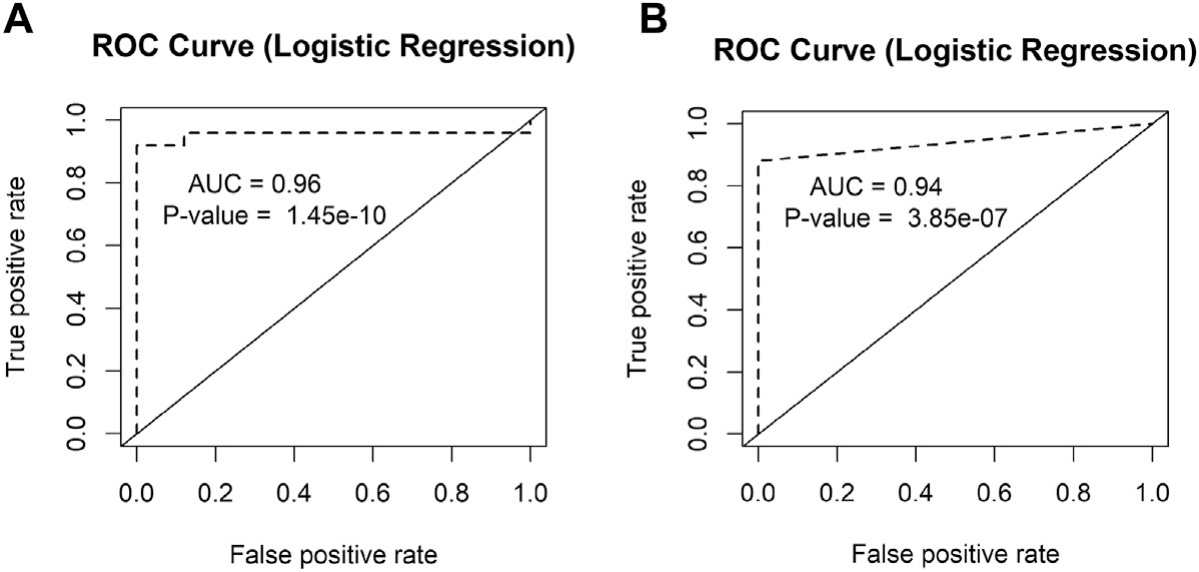
ROC analysis of the candidate circRNAs. The AUC values of the ROC curves of **(A)** circCEACAM5 and **(B)** circCOL1A1 represented the accuracy of these circRNAs in distinguishing GC and normal patients.

We also performed the receiver operating characteristic (ROC) curve analysis for our two candidate circRNA biomarkers circCEACAM5 and circCOL1A1 (Figures 8A,B). The area under the ROC curves (AUC) for these two circRNAs were significant (*p*-value<0.05). The AUC values 0.96 and 0.94 represented respectively the accuracy of circCEACAM5 and circCOL1A1 in distinguishing GC and normal patients.

### Gene Ontology Term and Kyoto Encyclopedia of Genes and Genomes Pathway Analysis

We constructed the PPI network from STRING database for the host genes of the DE circRNAs and selected the top 50 hub genes using cytoscape. To explore the functions of circRNAs, we performed the GO and KEGG pathway analyses for the top 50 hub genes of the DE circRNAs from the PPI network. We used the cut-off *p* < 0.05 for finding the significant pathways and GO terms. The GO term biological process analyses showed that some of the genes were enriched cell-cell adhesion, extracellular matrix organization, DNA repair, collagen catabolic process, collagen fibril organization, response to virus etc. The GO term molecular function analyses showed that some host genes were able to bind many kinds of molecules including ATP, protein, platelet-derived growth factor, DNA, microtubule, chromatin DNA, double-stranded RNA etc. Some other genes were enriched in cadherin binding involved in cell-cell adhesion, extracellular matrix structural constituent, microtubule motor activity etc. The GO term cellular component analyses showed that some genes were enriched in nucleus, nucleoplasm, cytosol, cytoplasm, mitotic spindle, basement membrane, mitotic spindle etc. The KEGG pathway analysis indicated that some host genes were enriched in several significant pathways including ECM-receptor interaction, PI3K-Akt signaling pathway, Focal adhesion, Cell cycle etc. The results of the significant GO term (top 30) and KEGG pathway analyses were provided in Tables 5, 6 respectively.

**TABLE 5:** Top 30 GO terms for the top 50 hub genes from the PPI networks of the host genes of the DE circRNAs.

GO term	GO name	No. of genes	*p*-value
**Biological process**			
GO:0,098,609	cell-cell adhesion	9	8.95E-07
GO:0,030,198	extracellular matrix organization	7	1.91E-05
GO:0,006,281	DNA repair	7	5.29E-05
GO:0,007,067	mitotic nuclear division	7	7.13E-05
GO:0,030,199	collagen fibril organization	4	1.86E-04
GO:0,009,615	response to virus	5	2.72E-04
GO:0,051,301	cell division	7	4.61E-04
GO:0,030,574	collagen catabolic process	4	8.08E-04
GO:0,007,051	spindle organization	3	9.36E-04
GO:0,006,260	DNA replication	5	9.91E-04
**Molecular Function**			
GO:0,005,524	ATP binding	18	4.83E-07
GO:0,098,641	cadherin binding involved in cell-cell adhesion	9	1.69E-06
GO:0,005,515	protein binding	41	1.57E-05
GO:0,048,407	platelet-derived growth factor binding	3	4.46E-04
GO:0,005,201	extracellular matrix structural constituent	4	9.66E-04
GO:0,003,677	DNA binding	13	0.002467
GO:0,008,017	microtubule binding	5	0.003076
GO:0,031,490	chromatin DNA binding	3	0.012302
GO:0,003,725	double-stranded RNA binding	3	0.013545
GO:0,003,777	microtubule motor activity	3	0.022588
**Cellular Component**			
GO:0,005,654	nucleoplasm	28	1.30E-10
GO:0,030,496	midbody	8	5.07E-08
GO:0,031,012	extracellular matrix	10	8.10E-08
GO:0,005,913	cell-cell adherens junction	9	2.14E-06
GO:0,005,829	cytosol	23	1.49E-05
GO:0,005,634	nucleus	30	1.71E-05
GO:0,072,686	mitotic spindle	4	1.81E-04
GO:0,005,876	spindle microtubule	4	2.24E-04
GO:0,005,737	cytoplasm	26	7.87E-04
GO:0,005,604	basement membrane	4	0.001251

**TABLE 6:** Significant pathways for the top 50 hub genes from the PPI network of the host genes of the DE circRNAs.

Pathway ID	Pathway description	No. of genes	*p*-value
hsa04512	ECM-receptor interaction	14	0.0000
hsa05146	Amoebiasis	10	0.0017
hsa04510	Focal adhesion	12	0.0031
hsa04151	PI3K-Akt signaling pathway	14	0.0062
hsa04974	Protein digestion and absorption	8	0.0091
hsa05145	Toxoplasmosis	8	0.0166
hsa04110	Cell cycle	8	0.0228
hsa05162	Measles	8	0.0273
hsa05160	Hepatitis C	8	0.0273
hsa00010	Glycolysis/Gluconeogenesis	6	0.0429
hsa01230	Biosynthesis of amino acids	6	0.0489

### Validation of the circRNAs by qReal-Time-Polymerase Chain Reaction

**FIGURE 9:**
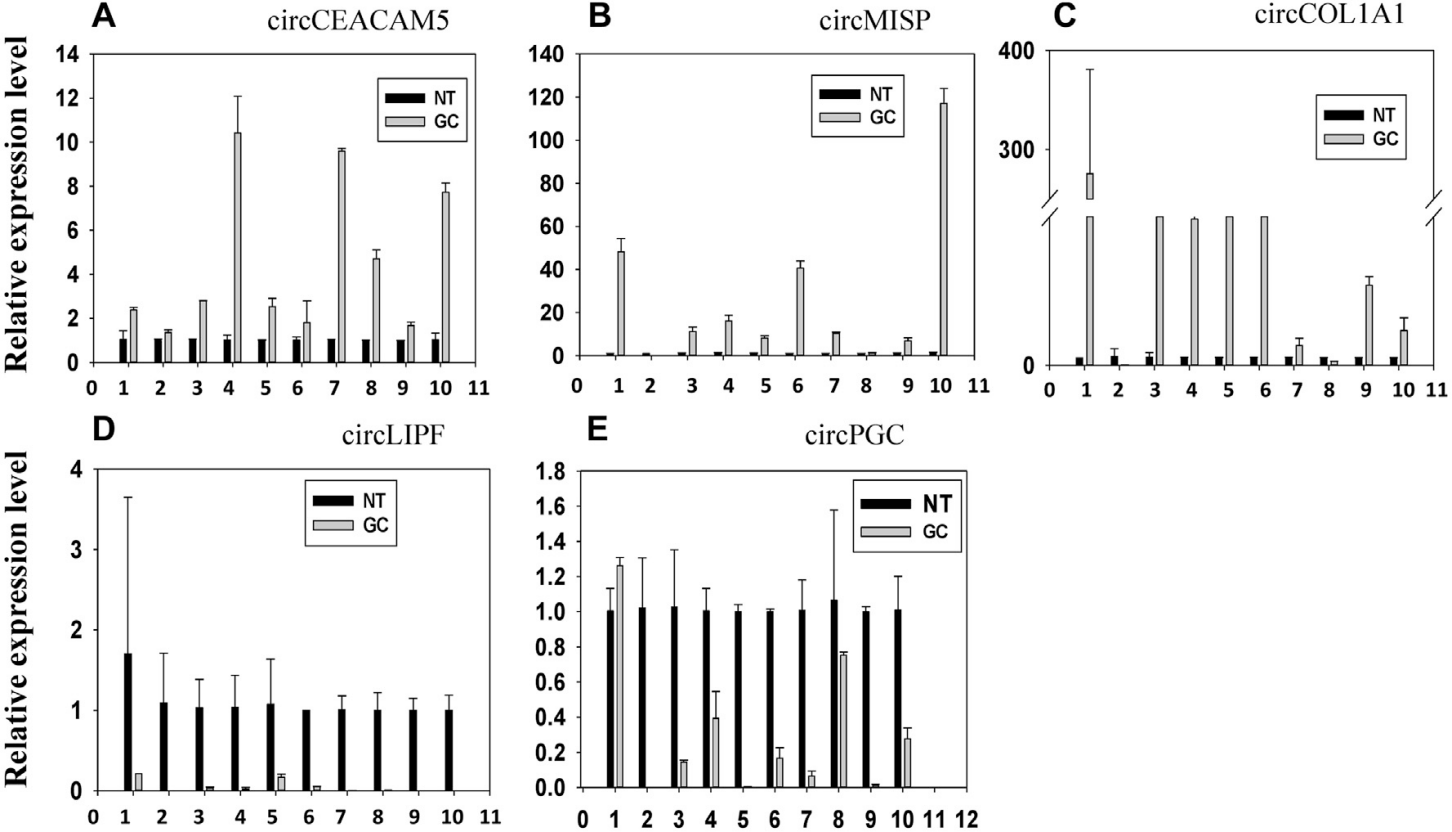
The relative expression level of top 5 up and downregulated circRNAs. The expression of **(A)** circCEACAM5, **(B)** circMISP, **(C)** circCOL1A1, **(D)** circLIPF and **(E)** circPGC in 10 paired gastric cancer (GC) specimens and the corresponding paired normal adjacent tissues (NT) was determined using real-time quantitative RT-PCR with divergent primers.

We selected 8 circRNAs (4 upregulated and 4 downregulated) for miRNA interaction analysis. These 8 circRNAs were DE in four (out of five) or more patients. Among these 8 circRNAs, we selected 5 circRNAs (3 upregulated and 2 downregulated) for experimental validation by qRT-PCR. The expression of these 5 circRNAs were shown in Figure 9. From Figure 9, we observed that the expression of circCEACAM5, circMISP, circCOL1A1 was upregulated in GC, which is consistent with the sequencing result. The expression of circLIPF and circPGC was decreased in GC in line with the sequencing result. Hence, the computational results for these 5 circRNAs were validated by the qRT_PCR experiments.

## Discussion

The role of circRNAs in human cancer is unrevealing gradually in recent years. In the present study, we predicted some circRNA biomarkers for gastric cancer through computational approach. We found a total of 45,783 circRNAs from 5 patients (10 samples). Then we performed DE analysis and found 4 upregulated and 7 downregulated circRNAs which were DE in four or more patients. Among these, we selected 8 circRNAs (4 upregulated and 4 downregulated) for miRNA interaction analysis.

The most important property of circRNA is known to function as miRNA sponges. As miRNA plays a crucial role in cancer progression, we explored the relationship between circRNAs and miRNAs through circRNA-miRNA-gene interaction network analysis. From the interaction network, we selected 5 hub circRNAs and found that the two circRNAs CircCEACAM5 and CircCOL1A1 were interacted with GC related miRNAs and their host genes were the important therapeutic and prognostic biomarker for GC. The circRNA CircCEACAM5 was interacted with 9 GC related miRNAs and among them miR-634 inhibits the proliferation, migration, and invasion of gastric cancer cell lines (Guo et al., 2018), miR-143-3p inhibits GC cell growth and induces apoptosis (Wu et al., 2013), and miR-429 acts as a tumor suppressor in GC cells (Zhang et al., 2016). The circRNA CircCOL1A1 was interacted with 5 GC related miRNAs for instances, miR-129-5p suppresses the proliferation of GC cells (Feng et al., 2020), miR-145-5p is able to inhibit the proliferation, migration and invasion of GC cells (Zhou et al., 2020), miR-501-5p promotes cell proliferation and migration in GC cells (Ma et al., 2020), miR-21-3p is closely related to GC and can be used to predict the prognosis of GC (Sun et al., 2021). We also performed the differential expression analysis of the targeted miRNAs of the circRNAs circCEACAM5 and circCOL1A1. We found that miRNAs miR-429, miR-21-3p and miR-145-5p significantly upregulated in GC compared to normal tissues. Thus, miR-429 might be the potential target of circCEACAM5 and, miR-21-3p and miR-145-5p might be the potential targets of circCOL1A1.

Another important property of circRNA is to regulate gene transcription (Meng et al., 2017). We performed the differential expression analysis of the parental genes of the 8 top up and down regulated circRNAs in GC using TCGA data. We found genes CEACAM5 and COL1A1 as upregulated in GC compared to normal tissues. These two genes were the key therapeutic and prognostic biomarkers for GC (Hu and Chen, 2012; Zhou et al., 2015; Zhaoxing Li et al., 2020). Thus, circCEACAM5 and circCOL1A1 have the potentials to regulate their parental gene transcription.

The GO term analysis of the parental genes of DE circRNAs showed that some host genes were involved in several important biological processes, molecular mechanisms and cellular components. The KEGG pathway analysis showed that some host genes were involved in several significant pathways such as ECM-receptor interaction, Focal adhesion, PI3K-Akt signaling pathway, Cell cycle etc. All of these pathways are involved in important mechanism of GC. ECM-receptor interaction pathway is identified as an important pathway associated with the progression of GC (Hu and Chen, 2012). Focal adhesion plays a key role in regulating cell survival, and proliferation, migration, and invasion of GC cells (Mao et al., 2021). The PI3K-Akt signaling pathway plays an important role in the development and progression of GC (Matsuoka and Yashiro, 2014). And a stronger enrichment of cell cycle pathway is found in GC (Saberi Anvar et al., 2018).

The host genes of these two circRNAs are also functionally related to GC. Gene CEACAM5 is a promising biomarker for prewarning and prognosis of GC (Zhou et al., 2015) and COL1A1 is considered as a potential biomarker for prognosis of GC (Hu and Chen, 2012; Zhaoxing Li et al., 2020). The two circRNAs circCEACAM5 and circCOL1A1 are DE in four or more patients (out of five), are substantially interacted with gastric cancer associated miRNAs, and the host genes of the DE circRNAs are involved in several gastric cancer related pathways. Furthermore the host genes of these two circRNAs are key therapeutic and prognostic biomarker for gastric cancer. The expression of these two circRNAs are also validated by qRT-PCR experiments. Hence, these two circRNAs might be potential biomarker for gastric cancer diagnosis and treatment.

## Conclusion

In the current study, we investigated potential circRNA biomarkers for GC through integrated analyses using 10 NGS samples collected from 5 patients. From differential expression and circRNA-miRNA-gene interaction analyses, we found two circRNAs circCEACAM5 and circCOL1A1 as DE and these circRNAs were interacted with GC related miRNAs. The host genes of these circRNAs were also the potential therapeutic and prognostic biomarkers for GC. The GO and KEGG pathway analyses revealed that some host genes of the DE circRNAs were enriched in several significant processes (biological, molecular and cellular) and enriched in several GC related pathways. These two circRNAs were also validated by the qRT-PCR experiments. Combining all the results, we can conclude that these two circRNAs circCEACAM5 and circCOL1A1 might play important role in the diagnosis and treatment of GC.

## Data Availability

The datasets presented in this study can be found in online repositories. The names of the repository/repositories and accession number(s) can be found below: China National Center for Bioinformation, accession PRJCA008130.
