# Corrigendum: Identification of circRNA biomarker for gastric cancer through integrated analysis

**DOI:** 10.3389/fmolb.2023.1249019

**Published:** 2023-07-04

**Authors:** Md. Tofazzal Hossain, Song Li, Md. Selim Reza, Shengzhong Feng, Xiaojing Zhang, Zhe Jin, Yanjie Wei, Yin Peng

**Affiliations:** ^1^ University of Chinese Academy of Sciences, Beijing, China; ^2^ Center for High Performance Computing, Joint Engineering Research Center for Health Big Data Intelligent Analysis Technology, Shenzhen Institutes of Advanced Technology, Chinese Academy of Sciences, Shenzhen, China; ^3^ Department of Statistics, Bangabandhu Sheikh Mujibur Rahman Science and Technology University, Gopalganj, Bangladesh; ^4^ Shenzhen Science & Technology Development Exchange Center, Shenzhen Science and Technology Building, Shenzhen, China; ^5^ Guangdong Provincial Key Laboratory for Genome Stability & Disease Prevention and Regional Immunity and Diseases, Department of Pathology, Shenzhen University School of Medicine, Shenzhen, China

**Keywords:** gastric cancer, circular RNA, computational approach, circRNA biomarker, circRNA-miRNA-gene interaction

In the published article, there was an error in the **Funding** statement. The grant number statement for the Shenzhen Basic Research Fund was displayed as “RCYX2020071411473419.” The correct grant number is “RCYX20200714114734194.” The correct Funding statement appears below.

“This work was partly supported by the National Key Research and Development Program of China under Grant No. 2018YFB0204403; Strategic Priority CAS Project XDB38050100; National Science Foundation of China under grant no. U1813203; the Shenzhen Basic Research Fund under grant nos JCYJ20200109114818703, RCYX20200714114734194, and JSGG20201102163800001; CAS Key Lab under grant no. 2011DP173015 (YW). We would also like to thank the funding support from the Youth Innovation Promotion Association, CAS to YW. This work is also supported by the National Foundation of Science (82172946) to ZJ; the National Foundation of Science (82173290) to XZ; Shenzhen Basic Research Fund (JCYJ20190808163801777) to YP.”

The authors apologize for this error and state that this does not change the scientific conclusions of the article in any way. The original article has been updated.

